# Shear bands and the evolving microstructure in a drying colloidal film studied with scanning µ-SAXS

**DOI:** 10.1038/s41598-018-31405-6

**Published:** 2018-08-28

**Authors:** Bin Yang, Nathan D. Smith, Andreas Johannes, Manfred Burghammer, Mike I. Smith

**Affiliations:** 1 School of Physics and Astronomy, University of Nottingham, Nottingham, NG7 2RD UK; 2 School of Pharmacy, University of Nottingham, Nottingham, NG7 2RD UK; 3 ESRF - European Synchrotron, CS40220, 38043 Grenoble, France

## Abstract

Shear localisation in thin bands is an important process involved in the plastic deformation of materials subject to stress. This process is often sensitive to the sample microstructure (amorphous/crystalline). Here we show using the scanning µ-SAXS technique, how these different microstructures influence the plastic deformations in a drying colloidal film. In crystalline samples, the presence of an ordering transition at the compaction front was directly identified through the development of a six-fold symmetry in the scattering pattern in 20 wt% samples. It is shown that plastic deformations in individual groups of particles during the compaction process can be tracked and measured in real time. Higher concentration suspensions were found to result in amorphous structures. The transition between crystalline and amorphous microstructures with initial particle concentration was also found to correlate with the appearance of shear bands. Through 2D spatial mapping of the local film structure, the presence of shear bands in the films was directly related to the microscale spatial variations in strain magnitude and compression direction. Our measurements also showed that shear bands lead to a reduction in the local particle volume fraction ~1–2%, indicating significant dilatancy.

## Introduction

Relating the plastic response of materials under an applied stress to their microstructure (crystalline, amorphous) is a problem of fundamental importance in a range of areas of materials science including granular^1^, complex fluids^2^ and metal alloys/glasses^3,4^. Under a uniaxial stress, shear can become localised within thin bands at ~45° to the direction of compression/tension. These shear bands can significantly modify a material’s properties, however they are still poorly understood. Whilst shear bands *can* form in crystalline materials, in bulk metallic glasses/alloys, shear localisation and the concomitant effects on material plasticity are believed to be strongly influenced by an amorphous microstructure^3,4^.

**Figure 1:**
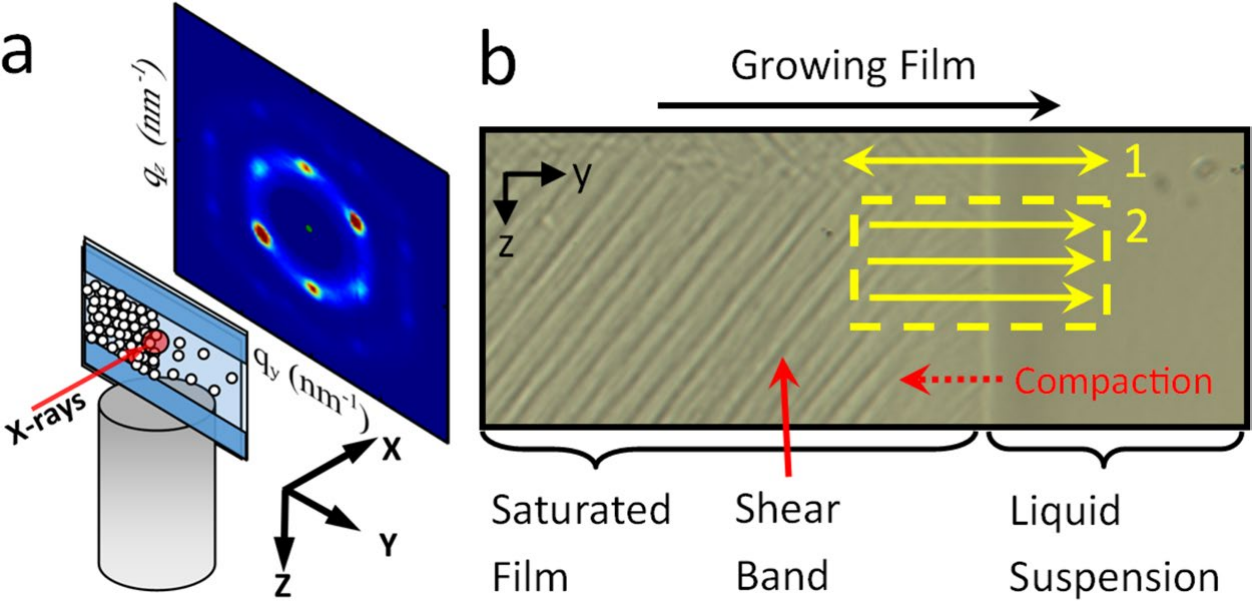
Scanning µ-SAXS setup. (**a**) Different concentration of AS-40 Silica Ludox were dried horizontally in cells constructed from 2 sheets of Mica with an 180 µm spacer. The microfocus beam (diameter 1 µm) probes a small region of the sample, resulting in a scattering pattern of the local microstructure. Scanning the sample through the beam, spatial variations in the local micro structure can be studied. (**b**) An optical microscope image of a drying sample showing the growth of shear bands which follow the growth of the film. Where the film first solidifies from the liquid suspension it gradually undergoes compaction. The sample can be scanned through the beam such that (1) single lines are scanned repeatedly or (2) a 2D box is measured.

Recently, it was shown that a drying colloidal film can also exhibit shear bands^5–7^. A colloidal film is created at the edge of a thin layer of drying suspension by evaporation, which causes a lateral flow of particles. This process is fundamental to the production of ceramic coatings, paint films and photonic crystals. As particles join the film from the suspension, they may undergo a transition from liquid-like to crystalline order^8^. However, it is also possible that drying induced stresses which result in compaction of the film may lead to an irreversibly aggregated and disordered network^8^. It is known that the final microstructure can be influenced by a variety of factors such as the rate of film formation^9^, particle charge^10^ and particle polydispersity^11,12^. Following this initial solidification into a film the particles undergo compaction (see Fig. 1a) leading to plastic deformations of the underlying microstructure^7,8^. However, with conventional techniques it is difficult to relate changes in film microstructure to much larger scale instabilities, such as shear bands.

Films of drying colloidal suspension have been widely studied using Small Angle X-ray Scattering (SAXS)^7,13–16^. Using this technique changes in the mean volume fraction^13^ and strain^7^ as the drying front moves can be inferred by collecting SAXS patterns from the relatively small region exposed to the incident beam. However, this region is still large compared to many of the structural features of interest (eg shear bands, cracks). Recently, Schroer *et al*.^17^ also performed SAXS measurements on *dry* films containing two different sizes of particle. Using a very small beam diameter (~400 nm) they were able to spatially map the phase separation of the two types of particle.

A few innovative optical microscopy studies have also made spatially resolved measurements of deformations near cracks^18^ and shear bands^19,20^. The direction of maximum compression was shown to vary locally by ~ ± 5° near shear bands in a colloidal film. However, spatially resolved measurements of the strain and volume fraction variations, which are used for example in theories of shear instabilities in glassy flows^21,22^ are still lacking.

In this study, the potential of the scanning micro-SAXS (µ-SAXS) technique at the European Synchrotron and Radiation Facility (ESRF) was exploited to understand the dynamic and spatial variations in local microstructure as films of colloidal silica formed. We directly observed the changes in ordering, for different film growth rates tracking small groups of particles as they underwent compaction in real-time. Significantly, we were able to measure key quantities such as the local strain, direction of maximum compression and volume fraction simultaneously, and show how these are related to the much larger scale shear banding instability.

Our sample cells consisted of two thick sheets of Mica separated by 180 µm thick spacers, containing a central channel which were filled with a silica suspension. The cell channels were oriented horizontally and perpendicular to the X-ray beam (see Fig. 1 and methods for details) and attached to a 2-axis high speed motorized stage. The beam size was restricted to 1 µm which is smaller than the length scale of the compaction process or shear banding. Two types of experiments are reported in this study: 1) single line scans, where the same line was scanned repeatedly perpendicular to the drying front (y axis) and 2) box scans, in which individual lines perpendicular to the drying front were scanned once before moving down to the next line, building up a box shaped series of measurements.

In our experiments we observed that the type of film microstructure that develops (crystalline or amorphous) is related to the film growth rate, and can be controlled by the choice of the initial concentration of the suspension. Initially we describe what can be learnt about the development and irreversible deformations that occur in samples of either crystalline or amorphous microstructure. Then we address how variations in the local microstructure can be related to the phenomenon of shear banding.

## Development and Deformations of a Film with crystalline microstructure

**Figure 2:**
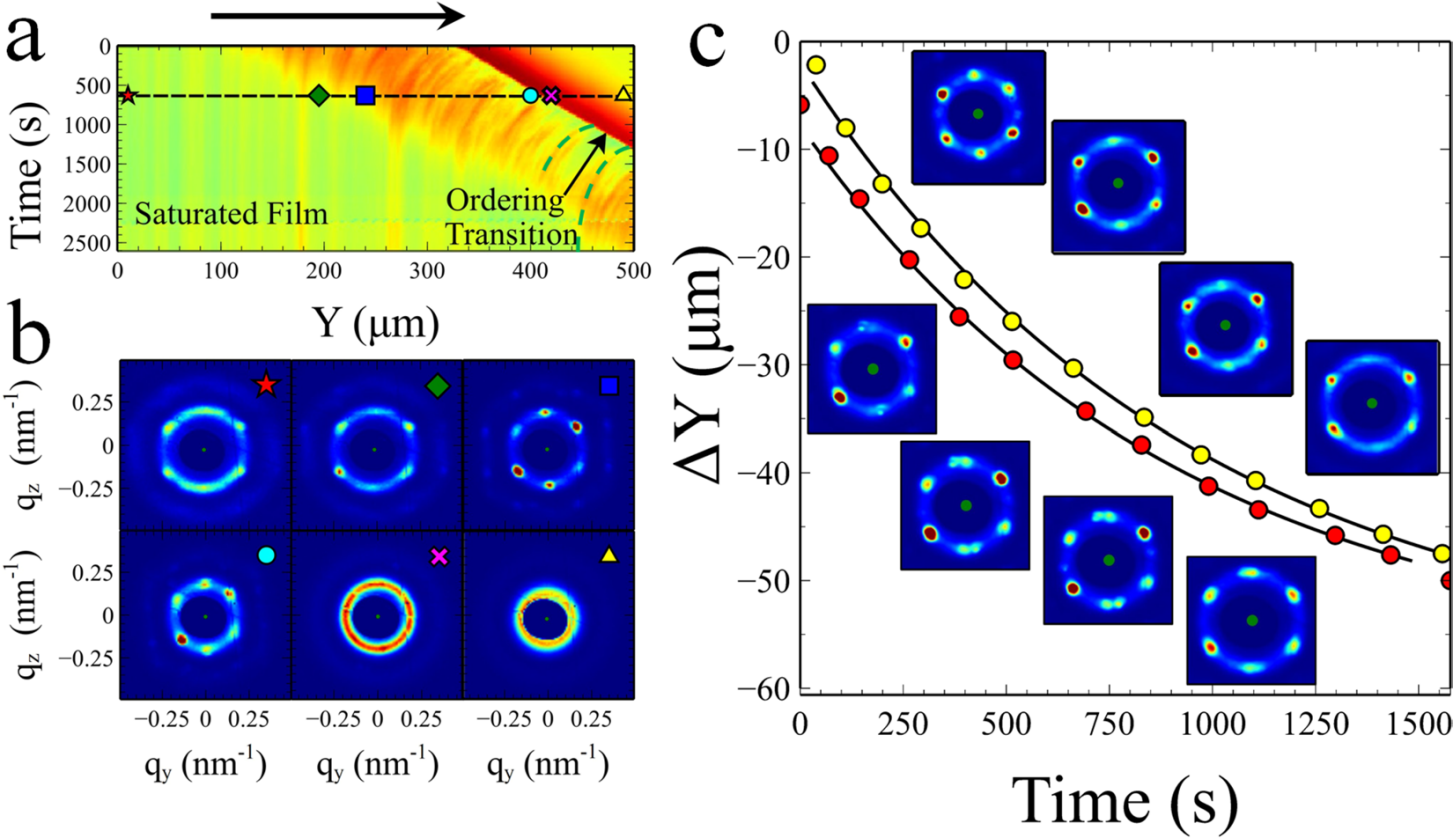
Scanning µ-SAXS of 20 wt% AS-40 colloidal silica (Sample thickness 180 µm). A 500 µm line was repeatedly scanned perpendicular to the drying front. (**a**) Graphical representation in which each pixel represents the integrated intensity of the scattering pattern collected at that position and time. The black arrow represents the direction of film growth (**b**) Representative scattering patterns from a single line scan at the positions shown by the corresponding symbols in (**a**) An ordering transition is seen to occur very abruptly when the particles are spaced ~34 nm apart, followed by a reduction in the degree of ordering as the patterns become anisotropic. (**c**) The motion of local regions of correlated intensity as a function of time (marked as two green curves in **a**). The “defects” and differences in spot intensity, observed in scattering patterns along these exponential paths show strong similarities. This indicates that the scanning µ-SAXS technique is able to follow the motion of a small group of particles as the film undergoes compaction.

Figure 2 shows data collected from a drying sample of 20 wt% silica in which a single line, perpendicular to the drying front, has been repeatedly scanned as the sample dries. Each pixel in Fig. 2a represents the integrated scattering intensity ($Q={\sum }_{qy,qz}I(q)$) of the corresponding scattering pattern collected at that location and time. In the µ-SAXS beam line at ESRF the scattering intensity is not normalised to the incoming beam intensity which may vary between experiments. The 2D maps of total scattering intensity should therefore be understood as images which enable the individual scattering patterns to be related to spatial features and not used without further calculation for quantitative comparison between different experiments. Examples of these scattering patterns at different locations are shown in Fig. 2b.

The µ-SAXS pattern at the far right of the image in Fig. 2a (), is an isotropic ring. This indicates a liquid dispersion of particles with an inter-particle separation d ~ 41 nm. Considering the measurements progressively further to the left, the particles become more concentrated whilst their spacing remains isotropic. This continues to be the case until the inter-particle spacing reaches a critical separation of ~34 nm. At this point, the scattering patterns change very suddenly (Δy <1 µm, Δt <25 s), from an isotropic ring, to spots that indicate a substantial degree of crystalline ordering () with either an FCC (111) or HCP (0001) structure. Using the µ-SAXS technique on such small areas of the film enables the direct observation of the particle ordering transition^8^.

To understand why the ordering transition occurs at this particular particle separation, we performed Dynamic Light Scattering measurements (DLS) on dilute suspensions, prepared via centrifugation from the 20 wt% experimental solutions (to preserve the same pH/ionic concentrations). The measured diameter of the silica particles was 34.6 ± 0.8 nm (ξ ~ −41mV ± 1) which is very similar to the inter-particle separation at which the ordering transition takes place (34.0 ± 0.5 nm). DLS measures the hydrodynamic diameter of particles, which encompasses the co-moving ionic double layers^16^ and is therefore larger than the contact diameter of the particle. This implies that the ordering transition occurs due to repulsive interactions between particles as the co-moving ions of the particles begin to overlap.

The crystalline scattering patterns observed in this region have some intriguing features. Firstly, the crystals are always oriented at the same angle, with the flat edge of the hexagonal pattern perpendicular to the direction of the drying. It seems likely that this orientation is set by the flow of evaporating liquid. Secondly, there is significant variability in the intensity of the individual spots in each scattering pattern. A minority of scattering patterns are also found to display faint additional spots, indicating the presence of defects in small regions of the film (Fig. 2c). A fact which enables us to study compaction in the films.

In Fig. 2a, following the ordering transition, curved lines of correlated intensity can be seen to trace a path in time covering ~40 µm in the y direction. In Fig. 2c we plot two of these curved paths whose location is shown by the green lines in Fig. 2a. The data points were obtained manually from the scattering intensity image in Fig. 2a (the red dataset has been offset by 5 µm for clarity). Repeated scanning of the same line in the film, results in multiple scattering patterns being obtained from the same local collection of particles as time passes. This makes it possible to track a particular set of particles within the film as they move or deform, providing their location can be identified in each subsequent line scan. Interestingly, the inset images in Fig. 2c show that variations in the peak intensities and anomalies in the scattering patterns outlined above, seem to persist along each curved path. However, when different locations along the ordering transition are compared, there are no such similarities. This indicates that each curved path represents the motion of a particular crystallised domain as it moves and deforms. The collective motion of the particle agglomerations forming these domains is also fitted well by a single exponential. Yang *et al*.^6^ tracked fluorescent particles embedded in a drying colloidal film, to measure the compaction during the initial stages of film formation. The motion of the tracer particles was shown to slow exponentially with distance from the solidification front. The curved paths in time and space therefore enable the dynamics of compaction in the y direction of a drying film to be followed in real time using the µ-SAXS technique.

Following the order transition, we observe that the mean particle spacing continues to decrease. At the point () the hexagonal pattern remains fairly symmetric and undistorted with a strain (measurement of which is described below) less than 2%. At the end of this region we observe a gradual transition (), in which the symmetry of the hexagonal packing gives way to an anisotropic structure and a significant strain ~5–6%. The elongated scattering pattern indicates compression in the drying direction and a partial loss of the particle ordering. However, spots in the scattering pattern are still clearly visible even a large distance into the film () indicating that some order remains quenched in the final film structure. The brighter side lobes at q_y_ = 0 of the scattering pattern at lower q_r_ values indicate that as the film is compressed in the drying direction it becomes relatively more ordered laterally across the drying front and less ordered in the direction of compression (q_y_)^7^.

This second gradual transition occurs over a relatively small region in the film and coincides with a mean particle spacing that is comparable to the contact diameter of the particles. While this transition is not as sharp as the ordering transition, it does seem to entail a relatively rapid change in the measured microstructure. It is particularly interesting that the loss of good crystalline ordering coincides with a rapid increase in the measured strain. This suggests that the presence of crystals to some degree stabilises the film against plastic deformations.

## Development and Deformations of a Film with Amorphous Microstructure

**Figure 3:**
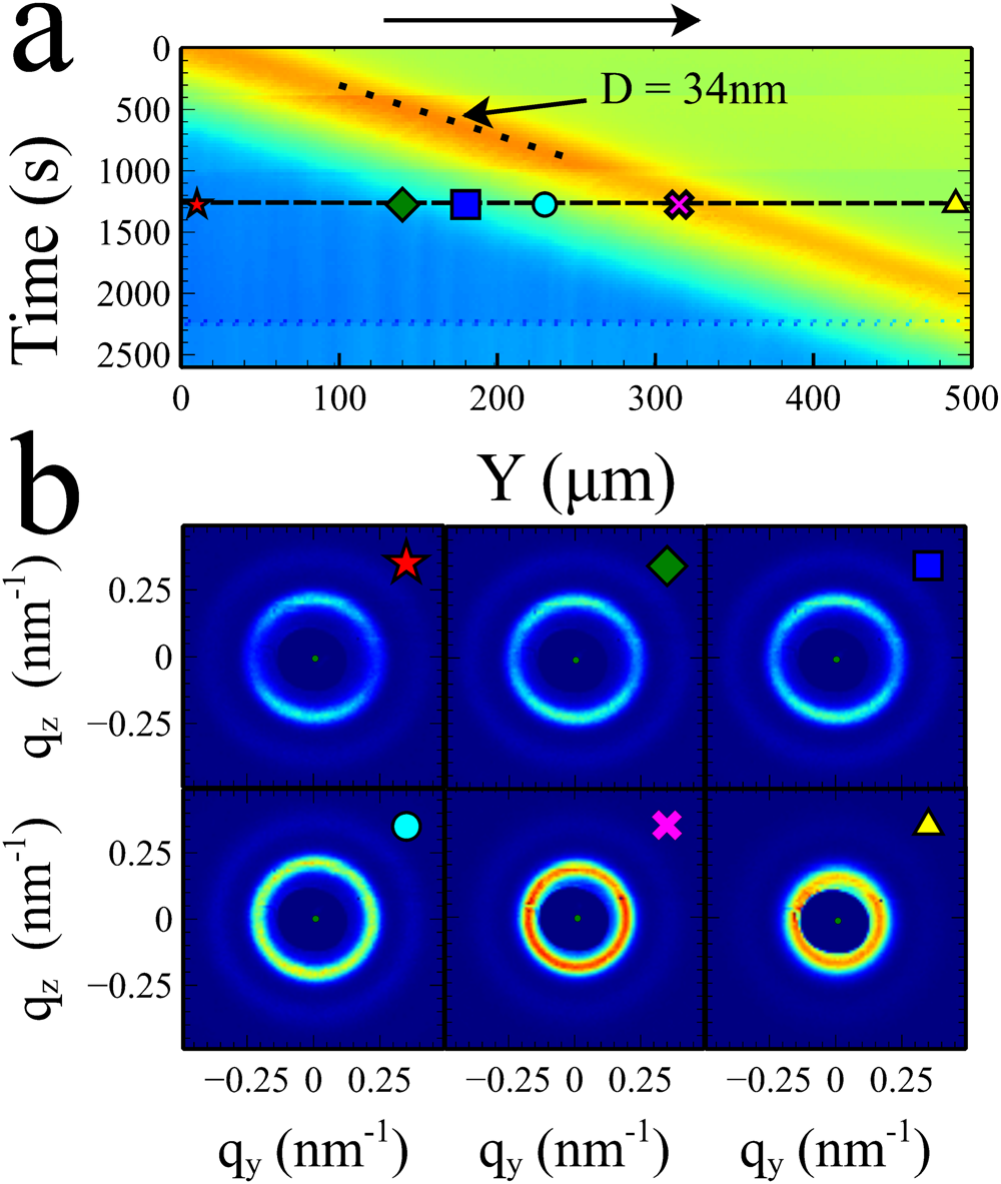
Scanning micro-SAXS of 30 wt% AS-40 colloidal silica (Sample thickness 180 µm). A 500 µm line was scanned repeatedly perpendicular to the drying front. (**a**) Graphical representation of the integrated intensity of each scattering pattern as a function of position and time. The diagonal dotted line indicates the position at which the particle spacing is ~34 nm apart, the same spacing at which the ordering transition takes place in Fig. 2. In contrast the scattering intensity varies smoothly in this regime. The black arrow represents the direction of film growth (**b**) Representative scattering patterns from a single line scan at the positions shown. No long-range ordering is observed but anisotropy develops gradually in the scattering pattern indicating the build-up of strain.

The velocity at which the compaction front grows into the cell can be measured directly from Fig. 2a through analysing the slope (u_y_ = Δy/Δt) of the ordering transition. This was found to be ~0.13 µms^−1^ for a 20 wt% sample (ϕ~0.10). Increasing the initial concentration of the silica particles in the suspension results in a faster film growth as the flux of particles swept towards the film by the evaporating liquid is increased. Figure 3 shows the same type of experiment shown in Fig. 2 but with an initial sample concentration of 30 wt% (ϕ ~ 0.16) where the velocity of film growth is ~0.24 µms^−1^.

Figure 3a shows the integrated intensity of the scattering patterns, examples of which are displayed in Fig. 3b. Unlike the 20 wt% sample in Fig. 2a, no ordering transition was observed. Instead the integrated intensity changes smoothly between a liquid (green) and the film (blue). In the solid film some small correlations in intensity, result in faint vertical stripes. However, despite attempts to vary the contrast and limits of the displayed data, we observed no similar curved lines in the region between liquid and solid as observed in Fig. 2a for the 20 wt% sample.

**Figure 4:**
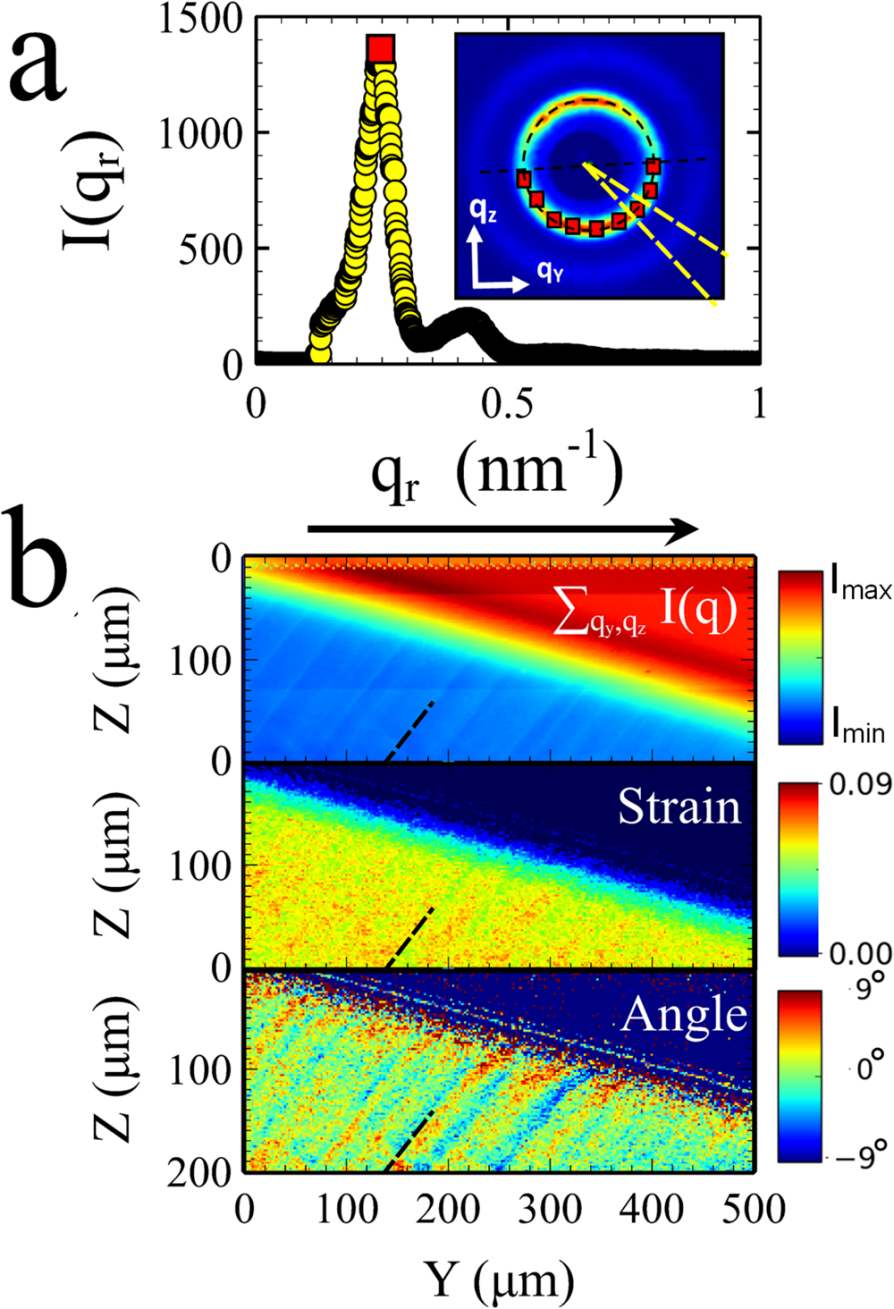
Relating shear banding to the local strains in the film. As the film starts to form scattering patterns are collected from 1µm^2^ areas of the film in different locations Y and Z. (**a**) Each scattering pattern is analysed by studying the radial scattering intensity profile from 90 segments at different azimuthal angles. The maximum of each intensity profile is extracted and its coordinates (q_z_,q_y_) are fitted to an ellipse which is used to calculate the strain magnitude and angle of the ellipse at a particular location of the sample. (**b**) 2D maps of the integrated scattering intensity, strain and ellipse angle, showing how these quantities vary on or near shear bands in the final film. The respective colour maps run from low (blue) to high (red) and covered the ranges 0–0.09 for the strain and ± 9° for the ellipse angle (blue = clockwise, red = anti-clockwise). The dotted black line in each image is in the same location and is added to enable easier comparison. The black arrow represents the direction of film growth.

The resulting scattering patterns and sample behaviour of the 30 wt% sample are qualitatively different from those observed in the 20 wt% samples. Firstly, none of the scattering patterns collected show any indications of crystalline spots. It is possible that the 30 wt% samples contain very small crystallites which are randomly orientated. However, these would have to be extremely small. In the 20 wt% samples, the crystals were also oriented preferentially with respect to the drying direction, which would make it unlikely that the ordering of crystallites would be azimuthally averaged into a uniform ring. Similar behaviour was also observed in samples made using an initial particle concentration of 40 wt% silica (ϕ ~ 0.23, see Fig. 4a). This indicates that the films formed from suspensions with a higher concentration are amorphous in structure.

The increased suspension concentration results in a dramatic change in the film microstructure. The main difference between the experiments for 20 and 30 wt% samples is predominantly the flux of particles arriving at the front of the growing film. Marín *et al*.^9^ showed that at the edge of a drying droplet of colloidal suspension one can observe a transition in the microstructure of particles, from ordered to disordered. The transition was found to depend on the deposition rate at which particles were added to the growing film. In essence, particles arriving at the growing film front do not have sufficient time to reach the equilibrium crystalline configuration before another layer of particles arrives, effectively quenching the dynamics and “freezing in” the amorphous liquid-like structure. This suggests our measurements bridge the critical particle flux required to disrupt the ordering transition.

Figure 3b shows that the scattering patterns obtained from particles during film formation are actually elliptical, with an elongated axis approximately in the drying direction (q_y_). Boulogne *et al*.^7^ showed that anisotropy in SAXS scattering patterns of drying colloidal films can be used to infer the deviatoric strain that develops in the film, ${\epsilon }=\frac{2}{3}(\frac{{q}_{z}}{{q}_{y}}-1)$. However, to assess the maximum magnitude of the strain ($|{\epsilon }|$), the ratio of scattering vectors needs to be the major and minor axes (q_major_, q_minor_) of the scattering ellipse. In our experiments, these are not always aligned precisely with the principal axes z, or y the direction of drying. Hence we define the maximum strain magnitude $|{\epsilon }|=\frac{2}{3}(\frac{{q}_{major}}{{q}_{minor}}-1)$. To accurately measure the ellipticity we implemented fitting routines in python in which each scattering pattern was split into 90 arc segments (see Fig. 4a). The maximum of the radial scattering pattern, extracted from each segment was located and then the coordinates (q_y_,q_z_) of these maxima were fitted to an ellipse. This enabled us to extract the major and minor axes of the scattering pattern ellipse and the angle (θ) between the major axis and the y axis (positive angles are defined as anti-clockwise from the y axis.).

It is interesting, to compare the anisotropy of the scattering patterns in the presence and absence of crystal formation. In Fig. 4 the anisotropy begins to develop some distance before the final film structure is formed () and increases relatively continuously up to a maximum value ~ 5–6%. As discussed above, in the 20 wt% samples the crystals remain fairly isotropic () until the final aggregation step occurs (), which is relatively much further behind the initial stages of film formation. However, the final value of strain measured in each case in the static saturated film is comparable. It appears therefore that the presence of the crystalline order in the film confers some additional stability/delays the distortion of the film micro-structure. In this context it is particularly intriguing that we observed a correlation between the absence of crystals from the scattering patterns and the presence of shear-banding in the final films. There is some indication from the literature that some other colloidal systems can form shear bands in the presence of crystals^23^. However, it implies that the crystalline/amorphous microstructure affects the development of plastic deformations in general and shear bands in particular, in agreement with observations from other systems such as bulk metallic glasses^3,4^.

## Relating Sample Microstructure to the Shear Banding Instability

**Figure 5:**
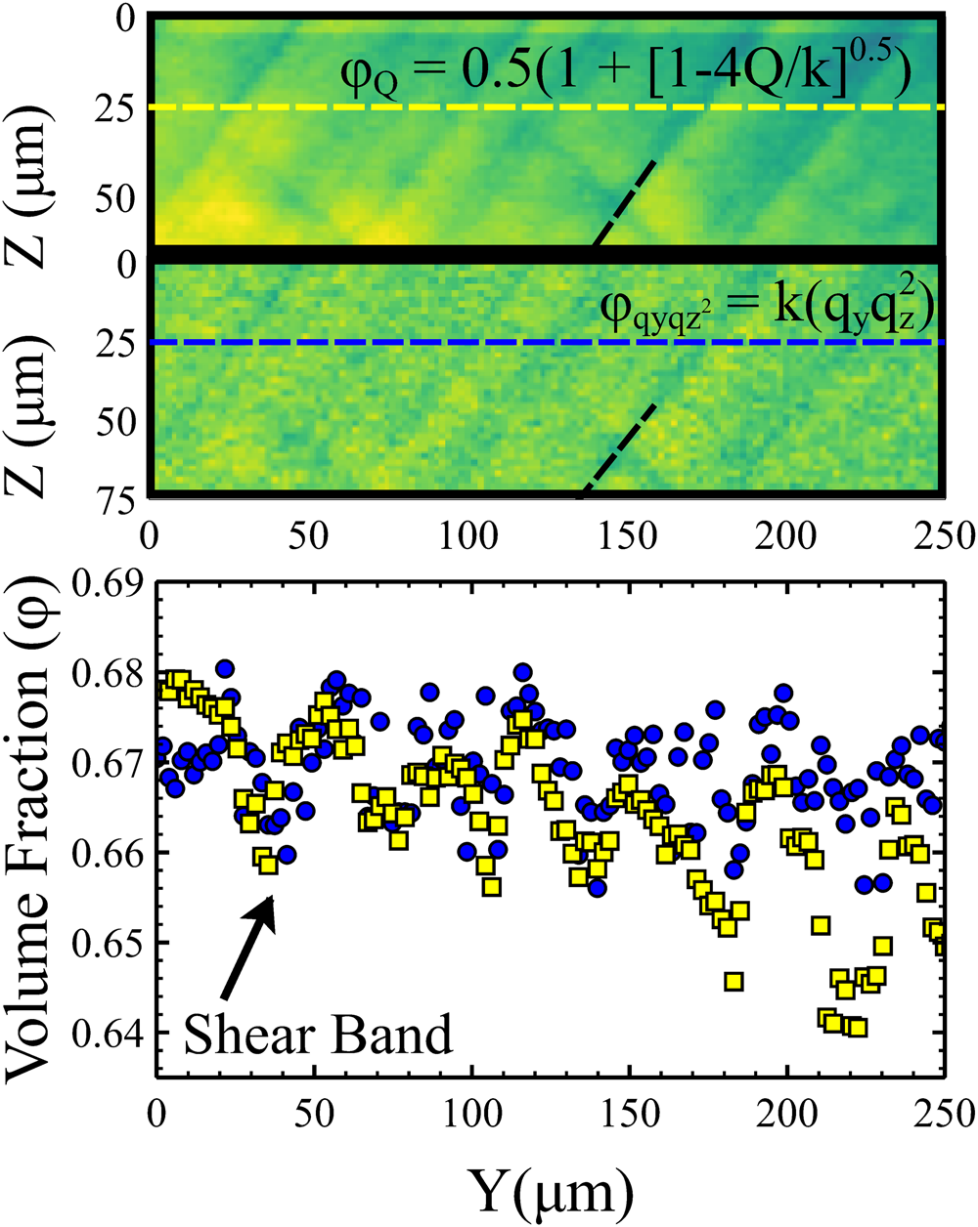
Changes in the local volume fraction due to shear band formation. The local volume fraction of the samples was calculated in two independent and complimentary ways. In both methods the maximum value of the volume fraction was assumed to be 0.68. The upper image was calculated from measurements of the integrated scattering intensity and the lower image was calculated from measurements of q_maj_ and q_min_ from the individual scattering patterns (see text for details). Each image shows similar periodic variations in the volume fraction which correlate well with the position of the shear bands. The dotted black line shown on each image is in the same place as the dotted black lines shown in Fig. 4b.

Unlike the data presented in Figs 2–5 represent multi-line scans in which a series of adjacent line scans are collected sequentially to build up a box shaped scan area. Using the µ-SAXS technique it is therefore possible to simultaneously measure the local particle microstructure *and* the much larger scale shear banding instabilities. This provides important new information about the spatial fluctuations in key properties (strain, volume fraction) around shear bands, rather than the coarse-grained averages probed by traditional SAXS.

In the top panel of Fig. 4b we plot the total integrated intensity of each scattering pattern at its corresponding location in the film for a drying sample of 40 wt% silica suspension. The scan was taken from top to bottom, with the fast scan direction along the y axis. As the liquid evaporates the film (blue) grows into the dilute suspension (red). Careful analysis of the elliptical scattering patterns, as outlined above, enables the quantitative study of the local variations in strain magnitude and direction (see bottom two images Fig. 4b).

Our results indicate that the magnitude of the strain in the final film varies between ~4 (green) and 7.5% (red). Bands of high and low strain are seen to alternate in a way that is commensurate with the observed shear bands in the integrated intensity image. In similar studies of a 30 wt% sample we also observed similar alternating values of the strain but with slightly smaller differences in the high and low strain values Δ|ϵ| ~ 2% (see Supplementary Fig. 1).

In addition to variations in the magnitude of the strain, our measurements show that the direction of the major axis of the scattering pattern (and therefore the direction of maximum compression) varies by ~ ± 6° (red = anti-clockwise, blue = clockwise). Just as with the strain, the variations in the angle of maximum compression are also correlated with the position of shear bands. Comparing the strain magnitude and angle it is found that lower strain regions are rotated anti-clockwise with respect to the y axis and higher strain regions are rotated clockwise (in samples where the shear bands are in the opposite direction this situation is reversed).

In addition to studying how the strain varies spatially it is also possible to measure the volume fraction variations induced by the shear banding process. This can be done in two independent and complimentary ways, both of which are shown in Fig. 5. In the top image of Fig. 5 the density is calculated from the total measured scattering intensity. Since the sample cell thickness is fixed, Q (the total integrated scattering intensity) can be used to infer changes in particle volume fraction. $Q=k\phi (1-\phi )$ where k includes the scattering contrast ${\rm{\Delta }}{\rho }^{2}={({\rho }_{particles}-{\rho }_{liquid})}^{2}$ with contributions from both particles and liquid and ϕ is the particle volume fraction^13^. Assuming a maximum volume fraction in the film ~0.68^13^, we solve for k and obtain an expression for the volume fraction $\phi =0.5(1+{[1-4Q/k]}^{0.5})$. The second image in Fig. 5 shows a similar map of the volume fraction, for the same region of film but calculated from measurements of the major and minor scattering vectors in each elliptical scattering pattern. In this we follow the method described by Boulogne *et al*.^7^ in which $\phi =k({q}_{major}{q}_{minor}^{2})$. This method contains more scatter but is in broad agreement for both the position and magnitude of volume fraction variations. These independent and complimentary measurements of the sample volume fraction show that the shear bands result in periodic increases and decreases in volume fraction of ~1.8 ± 0.5%. In a 30 wt% sample (where the film grows more slowly) smaller fluctuations were observed in the volume fraction ~ 1.0 ± 0.3% (see Supplementary Fig. 2). These values are comparable to changes measured for shear banding in carefully controlled granular flows^24,25^.

**Figure 6:**
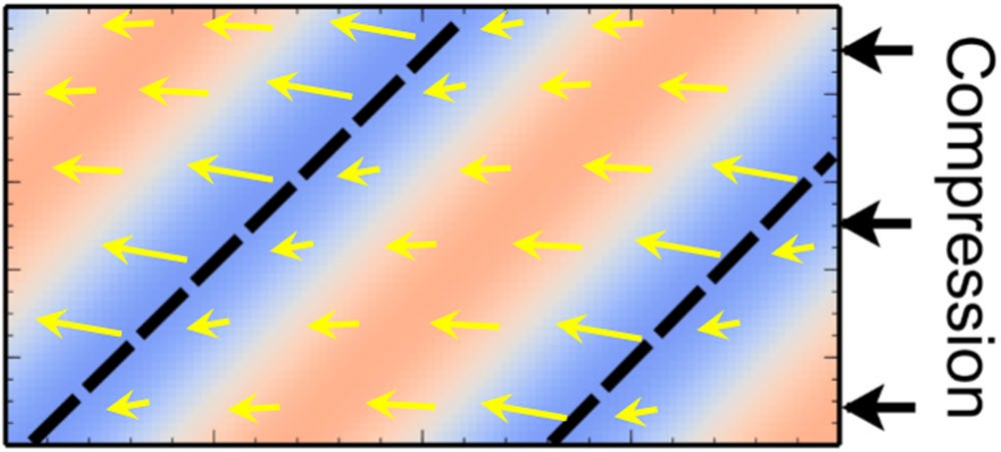
Schematic summary of local structural information in shear banded samples. The dotted lines are representative of the locations indicated by the dotted lines in Figs 4 and 5. The density exhibits a minimum at these locations (blue) and a maximum (red) in between. The arrows represent the magnitude and direction of the compressive strain. Changes from high to low strain occur relatively sharply at the location indicated by the dotted line.

To help the reader in comparing the spatial variations in strain and volume fraction, a black dotted line has been superimposed in the same place on the images of Figs 4 and 5. In Fig. 6 we also schematically summarise how the variations in density relate to the changes in the strain and angle of maximum compression. The location of the black dotted line corresponds to a minimum in the density where there is also a sharp change in the direction of flow/compression. This indicates that at this point there is significant local shear in the plane of the film. The narrow band at this location is therefore a region of enhanced flow.

Shear banding results from a bifurcation of the flow-rate in neighbouring bands of material. As the shear strain of closed packed particles increases there is often a corresponding reduction in density (dilatancy) which enables the system to flow^24–26^. The dilatancy in this narrow region would generate an inter-particle osmotic pressure that results in compression of the film ahead of the band and a corresponding increase in particle density. Behind the band the increased flow releases the strain as the direction of compression is rotated following the flow. These results demonstrate the presence of two co-flowing regions (ie shear banding) but also relate the spatially varying changes in local particle structure and density to the observed instability. Whilst it is difficult to make quantitative comparisons such fluctuations in density and strain appear similar to recent theoretical predictions for glass formers subject to compressive stresses at the boundaries^21,22^.

## Conclusions

In this study we have shown how the µ-SAXS mode at the ESRF beamline can be used to study the spatially varying dynamics of a drying colloidal film. Our work reveals the qualitatively different development and changes in film microstructure (crystalline or amorphous) which occur at different film growth rates. The spatially varying microstructure was related to the important process of shear banding, a detailed understanding of which affects many fields of materials science. It is our hope that such insights will enable theoretical progress in models of shear banding and inspire new experiments harnessing the capabilities of the µ-SAXS technique.

## Materials and Methods

### Sample and Cells

Sample cells were constructed using two sheets of Mica separated by a 180 µm thick spacer material (Meltonix^®^), which was cut to leave a ~ 5 mm wide central channel which was open at both ends. Suspensions were prepared via dilution of a monodisperse 40 wt% Ludox (As-40, Sigma Aldrich) with deionised water to the desired concentrations ranging from 20–40wt%. The cells were partially filled through capillary action by immersing one end temporarily in a small amount of the suspension. Once the liquid had filled the cells, they were mounted to a dual axis motorised sample stage, with the channel horizontal and perpendicular to the incident beam of X-rays. This was done to align the fast scan axis of the motorized stage perpendicular to the drying front.

### Beamline setup

The horizontal beam of incident X-rays (Energy ~13.9 keV) was restricted to a diameter ~1 µm. The resulting scattering patterns were recorded by an EIGER array detector (4 Megapixels, DECTRIS) at a distance of 0.535 m from the sample. At this distance, sufficient flux was obtained to record a single scattering pattern in ~50 ms. The relative position of the sample and beam were altered by raster scanning the sample through the beam using stepper motor based micro-positioners (100 nm resolution vertical, 500 nm resolution horizontal). In both cases points were collected at 2 µm intervals with, in the case of the box scans, each line also separated by 2 µm. Calibration of the detector was performed using the diffraction pattern of an Ag-Behenate crystal.

### DLS measurements

Dynamic Light Scattering measurements were performed using a Zetasizer nano ZS from Malvern instruments (λ = 633 nm). A 20 wt% solution of the commercial silica nanoparticle suspension Ludox As-40 was centrifuged three times at 12000 rpm for 10 hours, retaining the low particle concentration supernatant each time. This resulted in a low concentration particle solution, suitable for DLS which preserved the same pH/ionic concentration used in the SAXS experiments.

## Electronic supplementary material

Supplementary Information
