# Supplementary material for: Shear bands and the evolving microstructure in a drying colloidal film studied with scanning µ-SAXS
Source: Sci Rep. 2018 Aug 28;8:12979. doi: 10.1038/s41598-018-31405-6 (PMC6113273; doi:10.1038/s41598-018-31405-6)
Supplement: Supplementary file 1 — Supplementary Information [file 41598_2018_31405_MOESM1_ESM.pdf]

Supplementary Information: **Shear bands and the evolving microstructure in a drying colloidal film studied with scanning μ-SAXS**

Bin Yang,[1,2] Nathan D. Smith,[1] Andreas Johannes,[3] Manfred Burghammer[3] and Mike I. Smith[1]*

[1]School of Physics and Astronomy, University of Nottingham, Nottingham, UK, NG7 2RD

[2]School of Pharmacy, University of Nottingham, Nottingham, UK, NG7 2RD

[3]ESRF - European Synchrotron, CS40220, 38043 Grenoble, France

*Corresponding author: mike.i.smith@nottingham.ac.uk

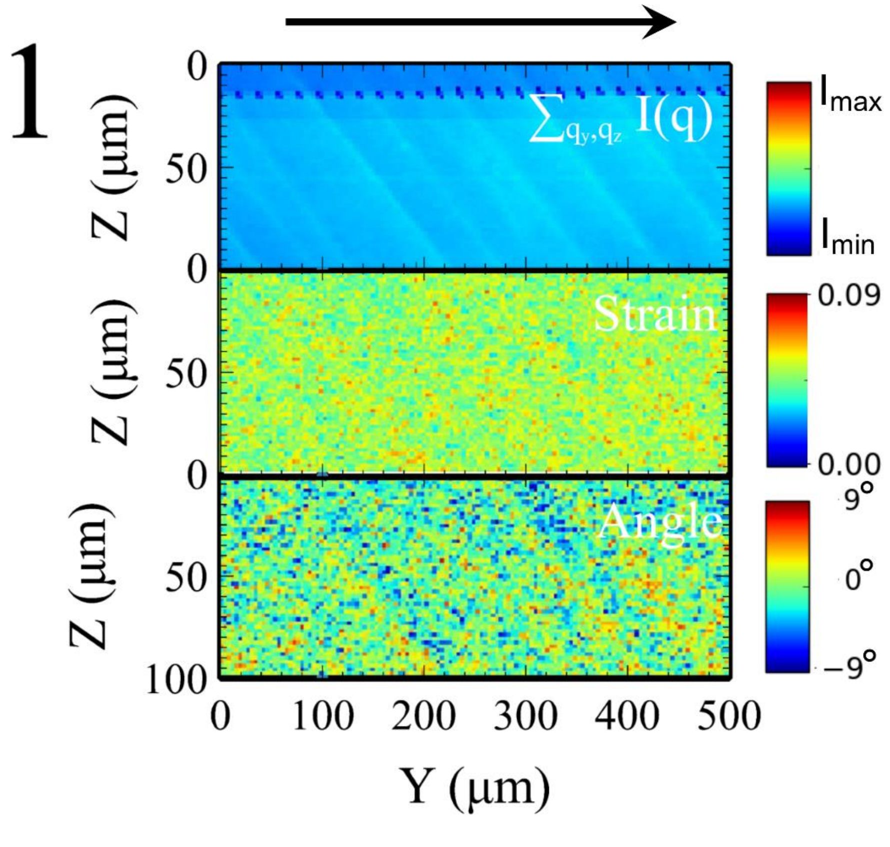

Supplementary Figure S1 – A map of the strain in the saturated film of a 30wt% sample of AS-40 Ludox. The color maps are the same as those used in figure 4b of the main article. The alternating pattern of strain follows that of the shear bands in the sample although the magnitude of the strain and its fluctuations are slightly smaller than in the 40wt% sample.

2

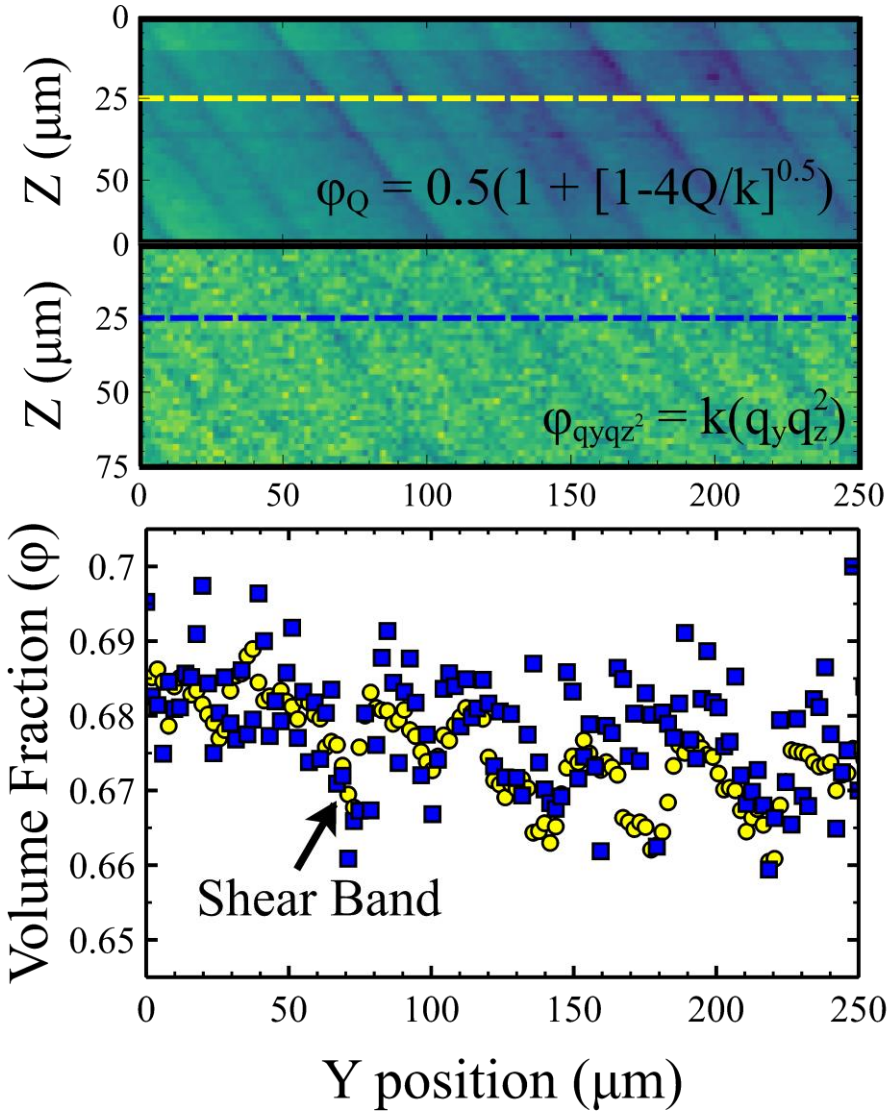

Supplementary Figure S2 – A map of the spatially varying volume fraction for a 30wt % AS-40 Ludox film in the same region of sample shown in supplementary figure S1. The fluctuations in the volume fraction are commensurate with the shear bands in the sample. The magnitude of the changes is ~ 1% +/- 0.3% which is slightly smaller than the 40wt% sample (see figure 5 main article).
